# Exometabolite-Based Antimicrobial Formulations from Lactic Acid Bacteria as a Multi-Target Strategy Against Multidrug-Resistant *Escherichia coli*

**DOI:** 10.3390/antibiotics14090851

**Published:** 2025-08-22

**Authors:** Gabriela N. Tenea, Diana Molina, Yuleissy Cuamacas, George Cătălin Marinescu, Roua Gabriela Popescu

**Affiliations:** 1 Biofood and Nutraceutics Research and Development Group, Faculty of Engineering in Agricultural and Environmental Sciences, Universidad Tecnica del Norte, Av. 17 de Julio s-21, Barrio El Olivo, Ibarra 100150, Ecuador; 2 Independent Research Association, 58 Timișului, Sector 1, 012416 Bucharest, Romania; catalin.marinescu@independent-research.ro (G.C.M.);; 3 Blue Screen SRL, 58 Timișului, Sector 1, 012416 Bucharest, Romania

**Keywords:** exometabolites, antimicrobials, multidrug resistant bacteria, lactic acid bacteria, cell-free supernatant

## Abstract

**Background/Objectives**: The global increase in multidrug-resistant (MDR) bacterial infections underscores the urgent need for effective and sustainable antimicrobial alternatives. This study investigates the antimicrobial activity of exometabolite-based formulations (ExAFs), derived from the cell-free supernatants (CFS) of native lactic acid bacteria (LAB) applied individually or in combination thereof, against MDR-*Escherichia coli* strain L1PEag1. **Methods**: Fourteen ExAFs were screened for inhibitory activity using time–kill assays, and structural damage to bacterial cells was assessed via scanning and transmission electron microscopy (SEM/TEM). The most potent formulation was further characterized by liquid chromatography–tandem mass spectrometry (LC–MS/MS) employing a Sequential Windowed Acquisition of All Theoretical Fragment Ion Mass Spectra (SWATH) approach for untargeted metabolite profiling. **Results**: Among the tested formulations, E10, comprising CFS from *Weissella cibaria* UTNGt21O, exhibited the strongest inhibitory activity (zone of inhibition: 17.12 ± 0.22 mm), followed by E1 (CFS from *Lactiplantibacillus plantarum* Gt28L and *Lactiplantibacillus plantarum* Gt2, 3:1 *v*/*v*) and E2 (Gt28L CFS + EPS from Gt2, 3:1 *v*/*v*). Time–kill assays demonstrated rapid, dose-dependent bactericidal activity: E1 and E10 achieved >98% reduction in viable counts within 2–3 h, at 1× MIC, while E2 sustained 98.24% inhibition over 18 h, at 0.25× MIC. SEM and TEM revealed pronounced ultrastructural damage, including membrane disruption, cytoplasmic condensation, and intracellular disintegration, consistent with a membrane-targeting mode of action. Metabolomic profiling of E10 identified 22 bioactive metabolites, including lincomycin, the proline-rich peptide Val–Leu–Pro–Val–Pro–Gln, multiple flavonoids, and loperamide. Several compounds shared structural similarity with ribosomally synthesized and post-translationally modified peptides (RiPPs), including lanthipeptides and lassopeptides, suggesting a multifaceted antimicrobial mechanism. **Conclusions**: These findings position ExAFs, particularly E10, as promising, peptide-rich, bio-based antimicrobial candidates for food safety or therapeutic applications. The co-occurrence of RiPP analogs and secondary metabolites in the formulation suggests the potential for complementary or multi-modal bactericidal effects, positioning these compounds as promising eco-friendly alternatives for combating MDR pathogens.

## 1. Introduction

Lactic acid bacteria (LAB) comprise a phylogenetically diverse clade of Gram-positive, facultatively anaerobic microorganisms, extensively utilized for their central role in the fermentation of both plant- and animal-derived substrates [1]. Beyond their fermentative functions, LAB are prolific producers of a complex array of extracellular metabolites, collectively referred to as exometabolites, accumulated in the cell-free supernatant (CFS). These include organic acids (e.g., lactic and acetic acid), hydrogen peroxide, diacetyl, reuterin, and a diverse spectrum of ribosomally synthesized and post-translationally modified peptides (e.g., bacteriocins) with potent antimicrobial properties [2,3]. These bioactive compounds contribute not only to microbial competitiveness and niche colonization but also play critical roles in preserving the safety and stability of food ecosystems [4].

In response to growing global concerns over antimicrobial resistance (AMR), the persistence of foodborne pathogens, and heightened consumer demand for natural, minimally processed foods, metabolites produced by LAB have emerged as promising and safe alternatives to conventional synthetic preservatives [5]. Their broad-spectrum activity, low toxicity, and minimal risk of promoting cross-resistance to clinical antibiotics make them promising candidates for integration into food-grade antimicrobial formulations [6].

In recent studies, we demonstrated that cell-free supernatants (CFS) and peptide extracts from native LAB strains isolated from Amazonian fruits effectively inhibit major foodborne pathogens, including both Gram-negative and Gram-positive species [7]. This inhibitory activity was mediated not only by pH-dependent acidification but also through peptide-based mechanisms with defined molecular targets [7]. Furthermore, we developed antimicrobial formulations from these peptide extracts that successfully suppressed *Citrobacter freundii* and *Staphylococcus aureus* in vitro [8]. More recently, we detected and characterized an MDR *E. coli* strain (L1PEag1) on the surface of commercially ready-to-eat fruits [9].

Despite the growing recognition of MDR bacterial contamination as a critical food safety hazard, the exploitation of probiotic-derived metabolites as targeted biocontrol agents in plant-based food matrices remains under-investigated. The heterogeneity of antimicrobial resistance determinants and persistence traits among pathogenic taxa underscores the necessity for the rational design of strain-specific antimicrobial formulations, followed by systematic validation under conditions simulating commercial production and supply chain environments [8].

In this context, the present study evaluates the inhibitory efficacy of exometabolite-based antimicrobial formulations (ExAFs), defined here as a combination of exometabolites from different LAB species (*Lactiplantibacillus plantarum* UTNGt2, *L. plantarum* UTNGt28L, and *W. cibaria* UTNGt21O) either in crude neutralized (pH 6.0) CFS form or in combination with exopolysaccharides (EPS) or stabilized labile compounds such as *Aloe vera* extract, prepared in ratios optimized for synergistic antimicrobial activity against MDR *E. coli* L1PEag1. Antimicrobial activity was assessed using time–kill kinetics and electron microscopy (SEM/TEM) to elucidate the impact on bacterial cell integrity. The most potent ExAF was further profiled by LC–MS/MS using a SWATH data-independent acquisition (DIA) approach, and structural chemical similarity analysis was performed to identify putative ribosomally synthesized RiPPs. The formulations were systematically evaluated to identify the most effective combinations for potential application in ex vitro biocontrol strategies against microbial contamination of fresh fruits. This study represents an important step toward the development of natural, pathogen-targeted interventions designed to enhance postharvest food safety and limit the spread of MDR bacteria throughout the food chain.

## 2. Results and Discussions

### 2.1. LAB Exometabolites Suppress Growth of MDR L1PEag1 in Co-Culture

This study assessed the antimicrobial efficacy of various ExAFs derived from the LAB-CFS against MDR *E. coli* strain L1PEag1. The LAB producer strains were previously investigated for their capacity to inhibit several foodborne pathogens [7,8]. Minimally processed CFS (pH 6.0), containing bioactive metabolites, offer a scalable and cost-effective antimicrobial approach. To enhance stability and functionality, selected ExAFs were formulated with LAB-derived EPS or *Aloe vera* extract (Table S1). EPS serves as a structural and protective matrix, potentially stabilizing labile antimicrobial compounds and prolonging their activity [10]. *Aloe vera* extract, rich in polysaccharides and known for its film-forming and wound-healing properties, was included to enhance bio adhesion and facilitate delivery [11,12]. Both additives are classified as Generally Recognized As Safe (GRAS) and are widely employed in pharmaceutical, food, and cosmetic applications, rendering them suitable for multifunctional delivery platforms.

**Figure 1:**
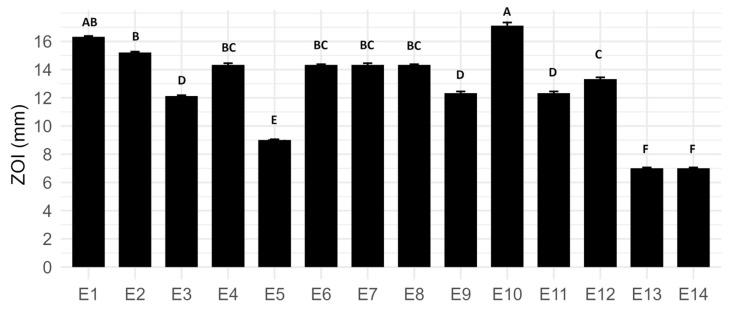
Zone of inhibition (ZOI) of *E. coli* L1PEag1 exposed to different LAB-derived ExAF formulations (E1–E14). Data represent mean (mm) ± standard deviation. Different letters above bars indicate statistically significant differences between treatments (*p* < 0.05, Tukey test).

Antimicrobial susceptibility assays revealed considerable variation in the inhibitory effects among the fourteen tested ExAF formulations (Figure 1). However, formulation E10, composed exclusively of CFS from *W. cibaria* UTNGt21O, exhibited the most significant inhibition against *E. coli* L1PEag1, with a zone of inhibition (ZOI) measuring 17.12 ± 0.22 mm (*p* < 0.05). This observation suggests that UTNGt21O produces highly potent and effective antimicrobials. Formulation E1, consisting of a 3:1 (*v*/*v*) mixture of CFS from *L. plantarum* strains Gt28L and Gt2, demonstrated strong inhibitory activity (ZOI: 16.32 ± 0.06 mm), indicating a potential synergistic effect when Gt28L is predominant. Interestingly, the addition of EPS in formulation E2 (CFS Gt28L combined with EPS from Gt2) enhanced antimicrobial efficacy (ZOI: 15.21 ± 0.06 mm) relative to the individual components (E11 and E12), supporting the hypothesis that EPS may improve the stability or diffusion of antimicrobial metabolites. Conversely, formulations containing *Aloe vera* extract (E3, E9, E13) exhibited reduced inhibitory activity, potentially due to sequestration or reduced permeability of active compounds within the viscous polysaccharide matrix.

**Figure 2:**
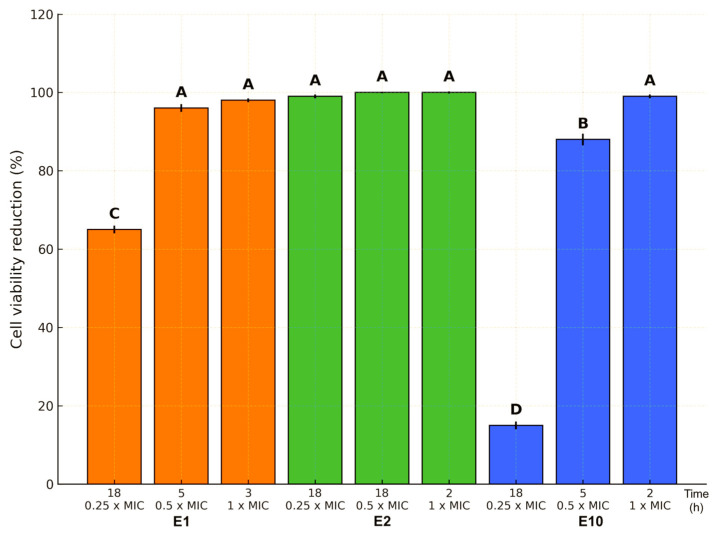
Reduction in L1PEag1 cell viability (%) by E1, E2, and E10 at 0.25×, 0.5×, and 1× MIC after different incubation times. E2 maintained complete inhibition across all concentrations, E1 achieved full inhibition at MIC within 3 h, and E10 displayed a delayed but strong effect at higher doses. Bars represent mean ± SE; different letters indicate significant differences (*p* < 0.05).

Moreover, the killing assay revealed a dose- and time-dependent bactericidal effect of ExAF formulations E1, E2, and E10 against MDR *E. coli* L1PEag1 (Figure 2). The three formulations (E1, E2, and E10) exhibited distinct antimicrobial potency profiles across sub-inhibitory (0.25× MIC, 0.5× MIC) and inhibitory (1× MIC) concentrations, with effects also influenced by exposure time. For E1, maximal killing (~100% cell viability reduction) was achieved at 0.5× MIC after 5 h and maintained at 1× MIC after 3 h, whereas 0.25× MIC required 18 h to reach only ~65% reduction, indicating both concentration- and time-dependence. E2 demonstrated the highest potency, achieving near-complete killing at all tested concentrations within 2–18 h, suggesting the presence of highly active compounds capable of rapid action, likely through potent membrane disruption or synergistic metabolic interference. In contrast, E10 showed minimal activity (~20% reduction) at 0.25× MIC after 18 h but displayed a steep increase in efficacy to ~90% at 0.5× MIC after 5 h and ~100% at 1× MIC after just 2 h, indicating a pronounced threshold effect where a critical concentration enables rapid bactericidal activity.

These results corroborate previous reports demonstrating potent bacteriolytic activity of PPGt21O from *W. cibaria* Gt21O, especially when combined with EPS from *W. confusa* Cys2-2, exhibiting strong antimicrobial effects against MDR pathogens [13]. Taken together, E1 and E10 demonstrate rapid-kill kinetics, whereas E2 sustained activity suggests suitability for prolonged-release applications. These formulations warrant further mechanistic and stability studies to advance eco-friendly, peptide-rich antimicrobials targeting MDR pathogens.

### 2.2. ExAFs Induced Morphological and Ultrastructural Cell Changes in E. coli L1PEag1

**Figure 3:**
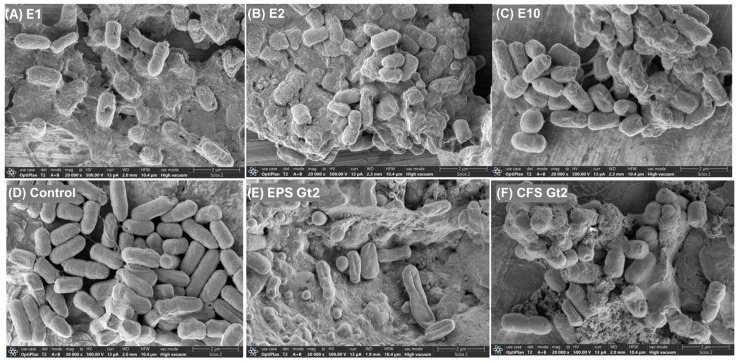
SEM micrographs of MDR *E. coli* L1PEag1 after treatment with different ExAFs. (**A**) E1 (CFS Gt28L: Gt2, 3:1, *v*/*v*); (**B**) E2 (CFS Gt28L + EPS Gt2, 3:1, *v*/*v*); (**C**) E10 (CFS Gt21O); (**D**) untreated control; (**E**) EPS Gt2 alone; (**F**) CFS Gt2 alone. Cells exposed to ExAFs (**A**–**C**) show surface irregularities and aggregation, while the control (**D**) retains normal rod-shaped morphology. EPS Gt2 (**E**) caused membrane damage and deformation, whereas CFS Gt2 (**F**) induced pronounced cell disruption and lysis, evidencing the antimicrobial effects of *L. plantarum*-derived metabolites. Magnification: 20,000×; scale bars: 2 µm.

**Figure 4:**
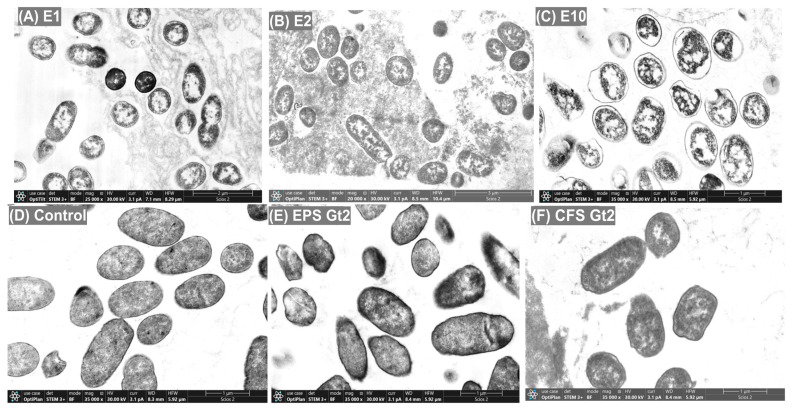
TEM analysis of MDR *E. coli* L1PEag1 following exposure to ExAFs. (**A**) E1 (CFS Gt28L: Gt2, 3:1, *v*/*v*); (**B**) E2 (CFS Gt28L + EPS Gt2, 3:1, *v*/*v*); (**C**) E10 (CFS Gt21O); (**D**) untreated control; (**E**) EPS Gt2; (**F**) CFS Gt2. Control cells (**D**) show intact morphology with preserved cell walls and homogeneous cytoplasm. Cells treated with E1, E2, and E10 (**A**–**C**) display extensive membrane disruption, cytoplasmic disorganization, and electron-lucent regions. EPS Gt2 (**E**) induced partial membrane rupture and cytoplasmic leakage, while CFS Gt2 (**F**) caused severe structural collapse and cell death. Magnification: 20,000×; scale bars: 1–3 µm.

The antimicrobial effects of ExAFs were further elucidated through SEM and TEM analysis, which revealed pronounced morphological and ultrastructural damage in treated bacterial cells compared to the untreated control (Figure 3 and Figure 4). SEM micrographs revealed distinct morphological differences between treated (Figure 3A–C,E,F) and untreated (Figure 3D) bacterial cells. In the untreated control group (Figure 3D), cells retain a typical rod-shaped morphology with smooth surfaces and intact membranes, indicative of healthy, undisturbed cells. In contrast, treatments with formulations E1, E2, and E10 (Figure 3A–C) resulted in marked structural alterations. Cells appeared deformed, exhibiting surface wrinkling, collapse, and irregular contours. Among these, E10 (Figure 3C) induced the most pronounced damage, with severe membrane disruption and cellular aggregation, supporting a potent bactericidal effect likely mediated by membrane-active metabolites such as phenolic acids and hydrophobic peptides. Treatment with EPS Gt2 (Figure 3E) caused moderate morphological alterations, including surface roughening and partial deformation, with cells embedded in an amorphous matrix. These observations suggest interactions between exopolysaccharides and the bacterial envelope, indicative of anti-biofilm activity or physical entrapment that contributes to antimicrobial efficacy. Likewise, the most drastic alterations were observed following exposure to the CFS Gt2 (Figure 3F), where extensive lysis, clustering, and membrane collapse point to the activity of secreted antimicrobial metabolites that likely disrupt membrane integrity and permeability. However, these results indicate that tropical-fruit-derived *L. plantarum* strains produce bioactive compounds with the capacity to compromise bacterial cell structures, supporting their potential as natural antimicrobial agents.

Complementary TEM micrographs confirmed these findings at the ultrastructural level: control cells exhibited intact membranes and well-preserved cytoplasmic content, while treated cells showed clear signs of membrane detachment, cytoplasmic condensation, and intracellular disorganization (Figure 4). In the control group (Figure 4D), cells exhibited typical hallmarks of viability, including evenly distributed cytoplasm, intact inner and outer membranes, and undisturbed nucleoid regions. In contrast, treatments with E1 and E2 (Figure 4A,B) led to evident cytoplasmic condensation, membrane detachment, and the presence of electron-lucent zones, indicating loss of membrane potential and intracellular leakage, hallmarks of pore-forming or ion-disruptive mechanisms. E10-treated cells (Figure 4C) displayed the most extensive damage, characterized by severely compromised membranes, cytoplasmic disintegration, and dense inclusion-like bodies, suggestive of rapid lysis or cell death. These alterations align with the synergistic activity of multiple antimicrobial metabolites, including lincomycin, phenolic acids, and the bioactive hexapeptide, reinforcing a multi-target bactericidal mechanism. EPS Gt2 treatment (Figure 4E) produced milder effects, including cytoplasmic granulation and partial membrane detachment, implying sub-lethal stress likely mediated by surface-binding exopolymers or metabolic interference. Instead, CFS Gt2 (Figure 4F) induced pronounced damage, including thinning of the cell wall, cytoplasmic condensation, and complete collapse of intracellular organization. These observations are consistent with previously reported effects of last-resort antibiotics such as colistin and imipenem on *Enterobacteriaceae*, which also cause severe membrane perturbation and cytoplasmic leakage [14]. The data strongly support the hypothesis that the ExAFs, particularly containing CFS from Gt21O, exert their antibacterial effect by targeting and destabilizing the bacterial envelope. This finding echoes earlier reports demonstrating the membrane-disruptive capabilities of PPGt21O against a range of Gram-negative and Gram-positive pathogens including *E. coli*, *Salmonella* spp., and *S. aureus* [8]. Together, the results indicate that the bioactive metabolites, particularly those in the E10 formulation, exert potent antimicrobial activity through a multifaceted mode of action, characterized by membrane destabilization, leakage of cytoplasmic contents, and extensive intracellular structural damage.

### 2.3. Metabolite Profiles Showed Compounds with Antimicrobial Properties

Formulation E10, derived from a chemically defined, single-strain CFS of *W. cibaria* UTNGt21O, was selected for detailed metabolite profiling due to its simplified matrix compared to the more complex multi-component formulations E1 and E2. LC–MS analysis detected 2093 precursor ions across positive and negative electrospray ionization (ESI) modes. Applying stringent filtering (MS1 tolerance 0.01 Da, MS2 tolerance 0.025 Da), 6 compounds were identified in ESI(+) and 16 in ESI(−) via accurate *m*/*z* matching and confirmed by tandem MS/MS spectral comparison (Table 1). The rapid bactericidal effect of E10 at sub-inhibitory concentrations (Table 2) indicates potent native antimicrobial metabolites.

Among identified compounds, lincomycin, a known bacterial protein synthesis inhibitor, and the antimicrobial hexapeptide Val–Leu–Pro–Val–Pro–Gln, a proline-rich antimicrobial peptide (PrAMP) class member, suggest multiple, possibly synergistic, antimicrobial mechanisms [15]; further analysis is required to validate this statement. PrAMPs possess structural flexibility allowing membrane interaction or intracellular targeting, inhibiting bacterial translation and/or forming transient pores, effective against multidrug-resistant pathogens [16]. This oligopeptides detection in both intracellular and extracellular fractions of *L. plantarum* UTNGt2 via integrated metabolomic and genomic analyses further supports its functional role in microbial defense [17]. Its presence across diverse LAB strains implies a conserved antimicrobial function. Loperamide, a phenylpiperidine antidiarrheal agent, exhibits antimicrobial potential through autophagy induction and immune modulation by altering intracellular calcium and suppressing proinflammatory cytokines TNFα, IL-6, and IFNγ [18]. It enhances intracellular control of *Mycobacterium tuberculosis* in alveolar macrophages, reducing inflammation-related tissue damage, positioning it as a candidate for drug repurposing in adjunctive therapy [18]. Structurally, loperamide shows moderate similarity to hominicin, a lanthipeptide from *Staphylococcus hominis* with potent activity against methicillin-resistant *Staphylococcus* strains [19]. However, the precise presence and structure of loperamide in our bacterial extract require further validation, as confirming its identity is critical to understanding its potential role in the observed antimicrobial effects. Daidzein, a soy isoflavone metabolite, demonstrates bacteriostatic activity against pathogens like *S. aureus* and synergizes with antibiotics to enhance efficacy [20,21]. Its co-occurrence with metabolites such as DL-4-hydroxyphenyllactic acid, dihydrocoumarin, and lithocholic acid in LAB fermenters suggests a multifaceted antimicrobial milieu suppressing pathogen growth and virulence [22]. Flavonoids chrysin and 3,7,4′-trihydroxyflavone, identified in E10, disrupt bacterial membranes and induce reactive oxygen species (ROS), likely contributing to antimicrobial effects against *E. coli* L1PEag1 [23]. Dihydrocoumarin, a benzopyranone derivative related to coumarins, inhibits microbial membranes, bacterial enzymes (DNA gyrase, topoisomerases), and quorum sensing, notably reducing *Pseudomonas aeruginosa* virulence and biofilm formation [24]. Overall, the detected metabolites not only corroborate the observed rapid bactericidal activity at low MICs but also underscore the promise of these bioactive compounds as leads for natural antimicrobial development.

**Table 2:** Metabolites with documented or potential antimicrobial activity.

Compound	Charge	Antimicrobial Role	Reference
Lincomycin	ESI (+)	Broad-spectrum antibiotic (protein synthesis inhibitor)	[25]
Chrysin	ESI (−)	Natural flavonoid with antibacterial and antifungal effects	[23]
Daidzein	ESI (−)	Isoflavone with synergistic effects with antibiotics	[20]
DL-4-Hydroxyphenyllactic acid	ESI (−)	Produced by LAB; contributes to antimicrobial activity	[22]
DL-p-Hydroxyphenyllactic acid	ESI (−)	LAB metabolite with mild antimicrobial effects	[22]
Dihydrocoumarin	ESI (−)	Inhibits bacterial quorum sensing, anti-virulence	[24]
3,7,4′-Trihydroxyflavone (5-Deoxykampferol)	ESI (−)	Flavonoid with antimicrobial activity and efflux pump inhibition	[26]
Loperamide	ESI (+)	Anti-virulence and antimicrobial activity against *Mycobacterium* spp.	[18]
Lithocholic Acid	ESI (−)	Secondary bile acid that inhibits gut pathogens	[27]
Palmitic acid	ESI (−)	Fatty acid with membrane-disrupting antimicrobial activity	[28]
*Val–leu–pro–val–pro–gln*	ESI (+)	Antimicrobial; identified in *L. plantarum* UTNGt2 with intracellular/extracellular action	[17]

### 2.4. ExAF Metabolite Profiling Reveals Structural Analogs to RiPPs with Antimicrobial Potential

Using similarity screening against the MIBiG RiPP database, several key ExAF metabolites demonstrated moderate to very high structural resemblance (Tanimoto coefficient ≥ 0.40) to various classes of ribosomally synthesized and post-translationally modified peptides (RiPPs), including lanthipeptides, lassopeptides, cyanobactins, glycocins, and thiopeptides (Table S2). These results align with emerging evidence that probiotics and food-grade microbes can biosynthesize bioactive peptides that mimic or enhance established antimicrobial pathways [29]. Among the ExAF constituents, the peptide-like compound *val–leu–pro–val–pro–gln* exhibited the highest similarity to known RiPPs, with Tanimoto scores reaching 0.81. It closely matched Zucinodin, Xanthomonin I, and Sphingonodin II, all well-characterized lassopeptides or lanthipeptides with potent antimicrobial properties [30]. Lassopeptides are particularly valued for their protease resistance, thermal stability, and mechanisms of action targeting DNA gyrase or RNA polymerase, features that may contribute to the rapid bactericidal activity observed in ExAF. Lincomycin, a known translation inhibitor, showed moderate similarity to RiPPs such as glycocins and lanthipeptides (Tanimoto scores: 0.41–0.45), suggesting a potential for synergistic action. Glycocins interfere with membrane-associated processes or cell wall biosynthesis [31], while flavonoids have been reported to disrupt membrane potential and enhance antibiotic uptake [32]. The co-occurrence of these compounds in ExAF may facilitate a concerted antimicrobial effect, targeting multiple bacterial systems simultaneously. Simple organic acids such as DL-hydroxyphenyllactic acid, traditionally considered metabolic intermediates, also showed moderate similarity to RiPP classes like phakellistatins. These acids may contribute to antimicrobial activity via pH alteration or induction of oxidative stress [33], indicating functional overlap with RiPPs and further supporting the concept of structural mimicry enhancing bioactivity. The alignment of lithocholic acid and palmitic acid with cyanobactins and phakellistatins reinforces previous findings that amphiphilic lipids and fatty acids can potentiate antimicrobial peptides by promoting membrane disruption or serving as adjuvants [34]. Even structurally distinct compounds such as loperamide, originally developed as an anti-diarrheal, have demonstrated antimicrobial properties, including efflux pump inhibition and membrane destabilization [35].

Based on these results, we suggest that the antimicrobial action of LAB-derived ExAFs against *E. coli* L1PEag1 may involve a multifactorial mode of activity mediated by structurally and functionally diverse metabolites. Potential contributors include ribosomally synthesized antimicrobial peptides, organic acids, flavonoids, phenolic compounds, and antibiotic-like molecules. The SEM and TEM analyses indicated pronounced morphological and ultrastructural changes in treated cells, such as disrupted membranes, condensed cytoplasm, and loss of internal organization, which are consistent with membrane destabilization playing a central role. The pronounced bactericidal effect observed at sub-MIC, particularly with the E10 formulation, raises the possibility that amphiphilic peptides and RiPP-like analogs facilitate membrane permeabilization, thereby enhancing the intracellular access of other active compounds. In parallel, molecules such as lincomycin and proline-rich peptides could interfere with protein synthesis by interacting with ribosomal targets, while flavonoids and phenolic acids might contribute through modulation of membrane integrity or other stress-related pathways; further targeted biochemical assays are required to demonstrate these statements.

## 3. Materials and Methods

### 3.1. Bacterial Strains and Culture Conditions

*L. plantarum* UTNGt28L (Gt28L) (BioProject: PRJNA1116628, BioSample: SAMN49560224), *L. plantarum* UTNGt2 (Gt2) (BioProject: PRJNA705232, BioSample: SAMN18053630), and *W. cibaria* UTNGt21O (Gt21O) (Genome Assembly: SRX8614718) were used. Fresh cultures were propagated on MRS (Man, Rogosa, and Sharpe) agar (Difco, Detroit, MI, USA) and incubated at 37 °C prior to use. The MDR *E. coli* L1PEag1, isolated from *Physalis peruviana* (uvilla), was cultured in LB (Luria-Bertani) broth (Merck Millipore, Burlington, MA, USA) as previously described [9]. All bacterial strains were preserved at −80 °C in 20% (*v*/*v*) glycerol stocks.

### 3.2. Preparation of ExAFs

CFS were obtained from overnight cultures of each LAB strain grown in MRS broth at 37 °C, following the protocol described by Garzón et al. [7]. After filtration through a 0.22 µm sterile syringe filter (Cat. #STF020025H, Chemlab Group, Washington, DC, USA) to remove residual cells, the CFS was neutralized to pH 6.0 with 5 M NaOH, lyophilized, and subsequently used in analytical assays [8]. For exopolysaccharide (EPS) isolation from Gt2, cultures (1 × 10^8^ CFU/mL) were grown in MRS broth supplemented with 20% (*w*/*v*) sucrose, and EPS was extracted via cold ethanol precipitation as previously described [36]. In certain formulations, a 5% *Aloe vera* extract was used as a cross-linking matrix in place of EPS. Antimicrobial activity was assessed by measuring the zone of inhibition (ZOI), defined as the diameter of the clear inhibitory area, in triplicate across three independent experiments. Complete formulation details and final concentrations are provided in Table S1.

### 3.3. Antimicrobial Activity Assay

The antimicrobial efficacy of each ExAF against *E. coli* L1PEag1 was evaluated in vitro using the agar well diffusion method [8]. Briefly, 100 μL of *E. coli* culture (~7 log CFU/mL in BHI broth) was mixed with 3.5 mL of 0.75% soft MRS agar and layered over Mueller–Hinton agar plates. After a 2 h pre-incubation at 37 °C to allow cell adherence, 100 μL of each formulation was applied to 6 mm wells cut into the agar surface. Plates were incubated at 37 °C for 48 h, and zones of inhibition were measured. All tests were conducted in triplicate, with results expressed as mean ± standard deviation.

### 3.4. Minimum Inhibitory Concentration (MIC) Assay of Selected ExAFs

The MIC was determined using a modified protocol adapted from Xiang et al. [37]. Antimicrobial activity was first expressed as titers in arbitrary units per milliliter (AU/mL). Titers were defined as the reciprocal of the highest two-fold dilution (2^n^) that inhibited visible growth of the indicator strain, with AU values calculated as AU = 2^n^ × (1000 µL/10 µL), where 10 µL represented the assay volume applied in the agar well diffusion test [38]. Growth inhibition was confirmed by the presence of clear zones ≥ 2 mm after 48 h incubation at 37 °C. For MIC determination, CFS ranging from 800 to 12,800 AU/mL were added to broth cultures of *E. coli* L1PEag1 and incubated for 24 h at 37 °C. Viable counts were determined by plating aliquots on agar, and the MIC was defined as the lowest CFS concentration achieving ≥ 90% growth reduction relative to the untreated control [39]. Among the tested formulations, the MIC corresponded to 1600 AU/mL.

### 3.5. Effect of ExAFs on L1PEag1 Cell Viability

Time–kill assay was performed as previously outlined by Wang et al. [40]. An overnight culture of *E. coli* L1PEag1 was adjusted to an initial concentration of 1 × 10^6^ CFU/mL and treated independently with each ExAF at 0.25×, 0.5×, and 1× MIC. Cultures were incubated at 37 °C, with untreated cells serving as negative controls. Bacterial viability was assessed at different intervals of time (0, 1, 2, 5, 7 and 18 h) post-treatment using the standard plate count method on BD Difco Plate Count Agar (Fisher Scientific, Hampton, NH, USA). The antimicrobial effect was quantified as the percentage reduction in viable counts, calculated as the log_10_(CFU/mL) difference between treated and untreated samples. A reduction exceeding 75% was considered highly efficient for the inhibitory action (statistically significant, *p* < 0.05). All assays were conducted in triplicate, and results are reported as mean ± standard deviation.

### 3.6. Scanning Electron Microscopy (SEM) of Treated Cells with Selected ExAFs

Surface morphological changes in *E. coli* L1PEag1 were analyzed using SEM. Both ExAF-treated and untreated cells were collected, washed, and resuspended in 1× phosphate-buffered saline (PBS). Samples were air-dried and fixed with 2.5% glutaraldehyde at 4 °C overnight. After triple phosphate buffer washes (5 min each) and a final rinse with distilled water, samples were dehydrated using an ethanol gradient (50–100%, 15 min per step), followed by critical point drying. Dried samples were mounted on graphite adhesive tape and sputter-coated with a ~24.5 nm layer of gold using a DENTON VACUUM Desk IV system (DENTON VACUUM, Austin, TX, USA). High-resolution imaging was conducted under high vacuum using a JSM-6490LV SEM (JEOL, Peabody, MA, USA) equipped with a secondary electron detector to assess cellular morphology and surface topography.

### 3.7. Transmission Electron Microscopy (TEM) of Treated Cells with Selected ExAFs

Ultrastructural alterations in *E. coli* L1PEag1 following exposure to ExAFs were examined by TEM. Bacterial cells in the exponential growth phase (1 × 10^6^ CFU/mL) were treated with 1× MIC of selected ExAFs for 6 h at 37 °C. Post-treatment, cells were chemically fixed, embedded, and sectioned according to established protocols. Ultrathin sections were mounted on copper grids and stained with 2% uranyl acetate followed by lead citrate (Sigma-Aldrich, St. Louis, MO, USA). A total of 10 randomly selected fields per treatment group were examined using a Tecnai G2 F20 transmission electron microscope (FEI Company, Hillsboro, OR, USA) to observe intracellular disruption and membrane integrity.

### 3.8. Metabolomic Profiling and Structural Similarity Analysis

Untargeted metabolomic profiling was carried out in accordance with the protocol described by Molina et al. [17]. Briefly, metabolite extracts (1 mg/mL) were subjected to centrifugation at 17,000× *g* for 15 min at 4 °C. Clar supernatants were analyzed using an AB SCIEX TripleTOF 5600+ mass spectrometer (Sciex, Concord, ON, Canada) integrated with a NanoLC 425 system (Eksigent, Dublin, CA, USA). Chromatographic separation was achieved on an Eksigent 5C18-CL-120 reverse-phase column (300 μm × 150 mm) using a 90 min linear gradient of 5–80% acetonitrile containing 0.1% formic acid, at a constant flow rate of 5 μL/min, and a column temperature of 55 °C. Mass spectrometric acquisition utilized a SWATH strategy employing 60 variable windows [17] in both positive and negative ESI modes. MS1 scans were acquired across *m*/*z* 100–1250 with an accumulation time of 150 ms, while MS2 scans ranged from *m*/*z* 100–2000 with a 30 ms accumulation time. Raw data were processed using MS-DIAL v5.3.240719, with deconvolution, peak alignment, and annotation based on spectral matching against curated MSP libraries (https://systemsomicslab.github.io/compms/msdial/main.html#MSP, accessed on 20 January 2025), at a 0.01 Da tolerance for MS1, and 0.025 Da tolerance for MS2. Only features with MS2 spectral similarity scores ≥ 70% were retained for further analysis. Annotated metabolite features were mapped to biochemical pathways using MetaboAnalyst 6.0, leveraging HMDB, PubChem, and KEGG databases to perform functional enrichment and classification [41,42]. For specialized metabolite analysis, particularly, putative ribosomally synthesized and post-translationally modified peptides (RiPPs), the processed LC–MS data were converted into SMILES format and analyzed via the RiPPMiner web platform [43]. Structural similarity between query compounds and known RiPPs was quantified using Tanimoto coefficient analysis [44], a molecular fingerprint-based method for binary encoding of chemical substructures. Compounds were classified into five similarity tiers: very low (0.0–0.2), low (0.2–0.4), moderate (0.4–0.6), high (0.6–0.8), and very high (0.8–1.0), with the latter indicating near-identical structural features. The ten highest-scoring candidate analogs were retained for downstream comparative and functional interpretation.

## 4. Conclusions

This study highlights that the exometabolites from *W. cibaria* UTNGt210 showed rapid, potent, and membrane-disruptive bactericidal activity against MDR *E. coli* L1PEag1. Metabolomic profiling revealed a complex repertoire of bioactive compounds—including bacteriocins, phenolic acids, flavonoids, and RiPP-like peptides such as Val–Leu–Pro–Val–Pro–Gln—likely responsible for synergistic, multi-target antimicrobial effects. These findings emphasize microbial-derived postbiotics as promising, mechanism-based alternatives to conventional antimicrobials, with applications in food safety, functional fermentation, and biomedical interventions.

## Figures and Tables

**Table 1:** List of metabolites detected by LC–MS/MS. Chemical charge, classification, chemical formulas, molecular masses, and their corresponding matches (or lack thereof) in various biochemical databases, including HMDB (Human Metabolome Database), PubChem, and KEGG.

Charge	Metabolite	Formula	Mass	Class	HMDB	PubChem	KEGG
ESI (−)	L-(-)-Malic acid	C_4_H_6_O_5_	133.02	Beta hydroxy acids and derivatives	HMDB0000156	222656	C00149
	L-Phenylalanine	C_9_H_11_NO_2_	165.06	Phenylalanine and derivatives	HMDB0000159	6140	C00079
	Eriodictyol-7-neohesperidoside	C_27_H_32_O_15_	595.16	Flavonoid-7-O-glycosides	-	-	-
	Tryptophan	C_11_H_12_N_2_O_2_	203.09	Indolyl carboxylic acids and derivatives	HMDB0000929	6305	C00078
	DL-4-Hydroxyphenyllactic acid	C_9_H_10_O_4_	163.04	1-hydroxy-2-unsubstituted benzenoids	-	-	-
	DL-p-Hydroxyphenyllactic acid	C_9_H_10_O_4_		Phenylpropanoic acids	HMDB0000755	9378	C03672
	Glimepiride	C_24_H_34_N_4_O_5_S	489.22	Benzenesulfonamides	HMDB0014367	3476	C07669
	Dihydrocoumarin	C_9_H_8_O_2_	147.05	3,4-dihydrocoumarins	HMDB0036626	660	C02274
	Chrysin	C_15_H_10_O_4_	253.06	Flavones	HMDB0036619	5281607	C10028
	Pravastatin	C_23_H_36_O_7_	423.24	Medium-chain hydroxy acids and derivatives	HMDB0005022	54687	C01844
	(2S,3S,4S,5R,6R)-6-[[(3S,6aR,6bS,8aS,14bR)-4,4,6a,6b,11,11,14b-heptamethyl-8a-[(2S,3R,4S,5S,6R)-3,4,5-trihydroxy-6-(hydroxymethyl)oxan-2-yl]oxycarbonyl-1,2,3,4a,5,6,7,8,9,10,12,12a,14,14a-tetradecahydropicen-3-yl]oxy]-3,4,5-trihydroxyoxane-2-carboxylic acid	C_42_H_66_O_14_	793.44	Triterpene saponins	-	-	-
	Daidzein	C_15_H_10_O_4_	253.04	Isoflavones	HMDB0003312	5281708	C10208
	3,7,4′-Trihydroxyflavone (5-Deoxykampferol)	C_15_H_10_O_5_	269.05	Flavonols	-	-	-
	Palmitic acid	C_16_H_32_O_2_	255.24	Long-chain fatty acids	-	-	-
	C10-LAS	C_16_H_26_O_3_S	297.16	Benzenesulfonic acids and derivatives	-	-	-
	Lithocholic Acid	C_24_H_40_O_3_	375.29	Monohydroxy bile acids, alcohols and derivatives	HMDB0000761	9903	C03990
ESI (+)	Loperamide	C_29_H_33_ClN_2_O_2_	477.23	Diphenylmethanes	HMDB0004999	3955	C07080
	Val–leu–pro–val–pro–gln	C_31_H_53_N_7_O_8_	244.12	Oligopeptides	-	-	-
	Lincomycin	C_18_H_34_N_2_O_6_S	407.22	Proline and derivatives	HMDB0015564	656509	C06812
	Tri(butoxyethyl)phosphate	C_18_H_39_O_7_P	399.24	Trialkyl phosphates	-	-	-
	Tetramethylscutellarein	C_19_H_18_O_6_	365.1	7-O-methylated flavonoids	HMDB0030575	96118	C14472
	Tryptophan	C_11_H_12_N_2_O_2_	188.07	Indolyl carboxylic acids and derivatives	HMDB0000929	6305	C00078

## Data Availability

The original contributions presented in this study are included in the article/Supplementary Materials. Further inquiries can be directed to the corresponding author.

## Supplementary Materials

The following supporting information can be downloaded at: https://www.mdpi.com/article/10.3390/antibiotics14090851/s1, Table S1: Description of ExAFs composition. Table S2: Predicted RIPPs based on metabolite chemical structure similarity of E10 metabolites with antimicrobial activity.
