# Supplementary material for: Exometabolite-Based Antimicrobial Formulations from Lactic Acid Bacteria as a Multi-Target Strategy Against Multidrug-Resistant Escherichia coli
Source: Antibiotics (Basel). 2025 Aug 22;14(9):851. doi: 10.3390/antibiotics14090851 (PMC12466617; doi:10.3390/antibiotics14090851)
Supplement: Supplementary file 1 [file antibiotics-14-00851-s001.zip › antibiotics-3812298-supplementary.pdf]

*Supplementary files*

# Exometabolite-Based Antimicrobial Formulations from Lactic Acid Bacteria as a Multi-Target Strategy Against Multidrug-Resistant ***Escherichia coli***

**Gabriela N. Tenea *[1], Diana Molina [1], Yuleissy Cuamacas [1], George Cătălin Marinescu [2,3] and Roua Gabriela Popescu [2,3]**

[1]Biofood and Nutraceutics Research and Development Group, Faculty of Engineering in Agricultural and Environmental Sciences, Universidad Tecnica del Norte, Av. 17 de Julio s-21. Barrio El Olivo, Postcode: 100150, Ibarra, Ecuador

[2]Independent Research Association, 58 Timișului, Sector 1, 012416 Bucharest, Romania.

[3]Blue Screen SRL, 58 Timișului, Sector 1, 012416 Bucharest, Romania.

* Correspondence: gntenea@utn.edu.ec

**Table S1.** Description of ExAFs composition.

| Code ExAFs | Description (concentration/ ExAFs and LAB strain code) |
|---|---|
| **E1** | **(1 x MIC) CFS Gt28L: CFS Gt2 (3:1, v/v)** |
| **E2** | **(1 x MIC) CFS Gt28L: EPS Gt2 (3:1, v/v)** |
| E3 | (1 x MIC) CFS Gt28L+ *Aloe vera* 5% |
| E4 | (1 x MIC) CFS Gt28L: CFS Gt2 (1:3, v/v) |
| E5 | (1 x MIC) CFS Gt28L: EPS Gt2 (1:1, v/v) |
| E6 | (1 x MIC) CFS Gt21O : CFS Gt28L (3:1, v/v) |
| E7 | (1 x MIC) CFS Gt21O : CFS Gt28L (1: 3, v/v) |
| E8 | (1 x MIC) CFS Gt21O : CFS Gt28L (1: 1, v/v) |
| E9 | (1 x MIC) CFS Gt21O + *Aloe vera* 5% |
| **E10** | **(1 x MIC) CFS Gt21O** |
| E11 | (1 x MIC) CFS Gt2 |
| E12 | (1 x MIC) CFS Gt28L |
| E13 | *Aloe vera* (5%) |
| E14 | (1 x MIC) EPSGt2 |

Legend: Gt28L: *L. plantarum* UTNGt28L; Gt2: *L. plantarum* UTNGt2; Gt21O: *W. cibaria* UTNGt21O; EPS: exopolysaccharides; CFS: cell free supernatant; MIC- minimum inhibitory concentration.

**Table S2.** Predicted RIPPs based on metabolite chemical structure similarity of **E10** metabolites with antimicrobial activity.

| Compounds | RiPP | Tanimoto score | Similarity | Interpretation | Category |
|---|---|---|---|---|---|
| Loperamide | Hominicin | 0.423077 | Moderate | Molecules share a fair number of features, possibly related but not very close. | Lanthipeptide |
| | Anacyclamide F10P AcyE | 0.391304 | Low | Some minor similarities exist, but the molecules are still structurally distinct. | Cyanobactin |
| | Zucinodin | 0.375 | Low | | Lassopeptide |
| | Cypemycin | 0.366667 | Low | | Linaridin |
| | Kawaguchipeptin B | 0.36 | Low | | Cyanobactin |
| | Carnocyclin A | 0.36 | Low | | Bacterial_head_to_tail_cyclized |
| | Anacyclamide A9P AcyE | 0.36 | Low | | Cyanobactin |
| | Streptomonomicin | 0.346154 | Low | | Lassopeptide |
| | Chaxapeptin | 0.346154 | Low | | Lassopeptide |
| | Caulonodin VI | 0.344828 | Low | | Lassopeptide |
| val-leu-pro-val-pro-gln | Zucinodin | 0.809524 | Very High/Identical | Molecules are highly similar or identical, with minimal structural differences. | Lassopeptide |
| | Xanthomonin I | 0.681818 | High | Molecules are closely related, likely with similar scaffolds or functional groups. | Lassopeptide |
| | Sphingonodin II | 0.68 | High | | Lassopeptide |
| | Prochlorosin 1.1 | 0.68 | High | | lanthipeptideB |
| | Venezuelin | 0.666667 | High | | lanthipeptideD |
| | Chaxapeptin | 0.666667 | High | | Lassopeptide |
| | Streptocollin | 0.653846 | High | | lanthipeptideD |
| | Paenicidin B | 0.653846 | High | | lanthipeptideA |
| | Syanodin I | 0.652174 | High | | Lassopeptide |
| | Ericin A | 0.642857 | High | | lanthipeptideA |
| Lincomycin | Hominicin | 0.451613 | Moderate | Molecules share a fair number of features, possibly related but not very close. | lanthipeptide |
| | Sublancin 168 | 0.45 | Moderate | | glycocin |
| | Nocathiacin I | 0.428571 | Moderate | | Thiopeptide |
| | Caulonodin IV | 0.419355 | Moderate | | Lassopeptide |
| | Xanthomonin II | 0.413793 | Moderate | | Lassopeptide |
| | Syanodin I | 0.413793 | Moderate | | Lassopeptide |
| | Sphingonodin I | 0.413793 | Moderate | | Lassopeptide |
| | Sphingonodin II | 0.40625 | Moderate | | Lassopeptide |
| | Sphingopyxin II | 0.4 | Moderate | | Lassopeptide |
| | Kawaguchipeptin B | 0.4 | Moderate | | Cyanobactin |
| Crysin | JBIR-83 | 0.458333 | Moderate | Molecules share a fair number of features, possibly related but not very close. | Thiopeptide |
| | Venturamide A | 0.454545 | Moderate | | Cyanobactin |
| | Nostocyclamide | 0.454545 | Moderate | | Cyanobactin |
| | Dendroamide A | 0.454545 | Moderate | | Cyanobactin |
| | JBIR-84 | 0.423077 | Moderate | | Thiopeptide |
| | Patellamide A patE4 | 0.416667 | Moderate | | Cyanobactin |
| | Patellamide A patE1 | 0.416667 | Moderate | | Cyanobactin |
| | Venturamide B | 0.416667 | Moderate | | Cyanobactin |
| | Tenuecyclamide D | 0.416667 | Moderate | | Cyanobactin |
| | Tenuecyclamide C | 0.416667 | Moderate | | Cyanobactin |
| Daidzein | Haliclonamide B | 0.454545 | Moderate | Molecules share a fair number of features, | Cyanobactin |
| | JBIR-83 | 0.434783 | Moderate | | Thiopeptide |
| | Venturamide A | 0.428571 | Moderate | | Cyanobactin |

| | | | | | |
|---|---|---|---|---|---|
| | Nostocyclamide | 0.428571 | Moderate | possibly related but not very close. | Cyanobactin |
| | Dendroamide A | 0.428571 | Moderate | | Cyanobactin |
| | JBIR-84 | 0.4 | Moderate | | Thiopeptide |
| | Bistratamide C | 0.4 | Moderate | | Cyanobactin |
| | Patellamide A patE4 | 0.391304 | Low | Some minor similarities exist, but the molecules are still structurally distinct. | Cyanobactin |
| | Patellamide A patE1 | 0.391304 | Low | | Cyanobactin |
| | Venturamide B | 0.391304 | Low | | Cyanobactin |
| DL-4-Hydroxyphenyl-lactic acid | Microphycin AL828 | 0.470588 | Moderate | Molecules share a fair number of features, possibly related but not very close. | Cyanobactin |
| | Hymenamide E | 0.470588 | Moderate | | Cyanobactin |
| | Hymenamide D | 0.470588 | Moderate | | Cyanobactin |
| | Phakellistatin 12 | 0.444444 | Moderate | | Cyanobactin |
| | Anacyclamide A7 AcyE | 0.421053 | Moderate | | Cyanobactin |
| | Stylissamide D | 0.411765 | Moderate | | Cyanobactin |
| | Stylissamide C | 0.411765 | Moderate | | Cyanobactin |
| | Stylissamide B | 0.411765 | Moderate | | Cyanobactin |
| | Stylisin 2 | 0.411765 | Moderate | | Cyanobactin |
| | Stylisin 1 | 0.411765 | Moderate | | Cyanobactin |
| DL-p-Hydroxyphenyl-lactic acid | Hymenamide E | 0.5625 | Moderate | Molecules share a fair number of features, possibly related but not very close. | Cyanobactin |
| | Phakellistatin 12 | 0.529412 | Moderate | | Cyanobactin |
| | Piricyclamide 7005 E3 PirE3 | 0.47619 | Moderate | | Cyanobactin |
| | Carnocyclin A | 0.47619 | Moderate | | Cyanobactin |
| | Haliclonamide D | 0.473684 | Moderate | | Cyanobactin |
| | Microphycin AL828 | 0.470588 | Moderate | | Cyanobactin |
| | Hymenamide D | 0.470588 | Moderate | | Cyanobactin |
| | PRENYLAGARAMIDE A | 0.45 | Moderate | | Cyanobactin |
| | Oscillacyclin | 0.45 | Moderate | | Cyanobactin |
| | Cyclonellin | 0.45 | Moderate | | Cyanobactin |
| Dihydrocoumarin | Stylissamide D | 0.466667 | Moderate | Molecules share a fair number of features, possibly related but not very close. | Cyanobactin |
| | Stylissamide C | 0.466667 | Moderate | | Cyanobactin |
| | Stylissamide B | 0.466667 | Moderate | | Cyanobactin |
| | Stylisin 2 | 0.466667 | Moderate | | Cyanobactin |
| | Stylisin 1 | 0.466667 | Moderate | | Cyanobactin |
| | Phakellistatin 9 | 0.466667 | Moderate | | Cyanobactin |
| | Phakellistatin 8 | 0.466667 | Moderate | | Cyanobactin |
| | Phakellistatin 7 | 0.466667 | Moderate | | Cyanobactin |
| | Phakellistatin 2 | 0.466667 | Moderate | | Cyanobactin |
| | Phakellistatin 1 | 0.466667 | Moderate | | Cyanobactin |
| 3,7,4'-Trihydroxyflavone (5-Deoxykampferol) | Haliclonamide B | 0.454545 | Moderate | Molecules share a fair number of features, possibly related but not very close. | Cyanobactin |
| | JBIR-83 | 0.434783 | Moderate | | Cyanobactin |
| | Venturamide A | 0.428571 | Moderate | | Cyanobactin |
| | Nostocyclamide | 0.428571 | Moderate | | Cyanobactin |
| | Dendroamide A | 0.428571 | Moderate | | Cyanobactin |
| | JBIR-84 | 0.4 | Moderate | | Cyanobactin |
| | Bistratamide C | 0.4 | Moderate | | Cyanobactin |
| | Patellamide A patE4 | 0.391304 | Low | Some minor similarities exist, but the molecules are still structurally distinct. | Cyanobactin |
| | Patellamide A patE1 | 0.391304 | Low | | Cyanobactin |
| | Venturamide B | 0.391304 | Low | | Cyanobactin |
| Lithocholic Acid | Phakellistatin 12 | 0.588235 | Moderate | Molecules share a fair number of features, | Cyanobactin |
| | Phakellistatin 13 | 0.555556 | Moderate | | Cyanobactin |
| | Hymenamide H | 0.555556 | Moderate | | Cyanobactin |
| | Axinellin C | 0.555556 | Moderate | | Cyanobactin |

| | | | | | |
|---|---|---|---|---|---|
| | Xanthomonin II | 0.55 | Moderate | possibly related but not very close. | Lassopeptide |
| | Sphingonodin I | 0.55 | Moderate | | Lassopeptide |
| | Kawaguchipeptin A | 0.545455 | Moderate | | Cyanobactin |
| | Microphycin AL828 | 0.529412 | Moderate | | Cyanobactin |
| | Hymenamide E | 0.529412 | Moderate | | Cyanobactin |
| | Hymenamide D | 0.529412 | Moderate | | Cyanobactin |
| Palmitic acid | Microphycin AL828 | 0.5 | Moderate | Molecules share a fair number of features, possibly related but not very close. | Cyanobactin |
| | Hymenamide D | 0.5 | Moderate | | Cyanobactin |
| | Hymenamide C | 0.466667 | Moderate | | Cyanobactin |
| | Hymenamide B | 0.466667 | Moderate | | Cyanobactin |
| | Anacyclamide A7 AcyE | 0.4375 | Moderate | | Cyanobactin |
| | Stylissamide D | 0.428571 | Moderate | | Cyanobactin |
| | Stylissamide C | 0.428571 | Moderate | | Cyanobactin |
| | Stylissamide B | 0.428571 | Moderate | | Cyanobactin |
| | Stylisin 2 | 0.428571 | Moderate | | Cyanobactin |
| | Stylisin 1 | 0.428571 | Moderate | | Cyanobactin |
